# Statistical methods for linking geostatistical maps and transmission models: Application to lymphatic filariasis in East Africa

**DOI:** 10.1016/j.sste.2020.100391

**Published:** 2022-06

**Authors:** Panayiota Touloupou, Renata Retkute, T. Déirdre Hollingsworth, Simon E.F. Spencer

**Affiliations:** a School of Mathematics, University of Birmingham, Birmingham, UK; b Department of Plant Sciences, University of Cambridge, Cambridge, UK; c Big Data Institute, Li Ka Shing Centre for Health Information and Discovery, Nuffield Department of Medicine, University of Oxford, Oxford, UK; d Department of Statistics, University of Warwick, Coventry, UK; e Zeeman Institute, University of Warwick, Coventry, UK

**Keywords:** Bayesian methods, Fine-scale spatial predictions, Linking maps with models, Lymphatic filariasis, Projections, Uncertainty

## Abstract

Infectious diseases remain one of the major causes of human mortality and suffering. Mathematical models have been established as an important tool for capturing the features that drive the spread of the disease, predicting the progression of an epidemic and hence guiding the development of strategies to control it. Another important area of epidemiological interest is the development of geostatistical methods for the analysis of data from spatially referenced prevalence surveys. Maps of prevalence are useful, not only for enabling a more precise disease risk stratification, but also for guiding the planning of more reliable spatial control programmes by identifying affected areas. Despite the methodological advances that have been made in each area independently, efforts to link transmission models and geostatistical maps have been limited. Motivated by this fact, we developed a Bayesian approach that combines fine-scale geostatistical maps of disease prevalence with transmission models to provide quantitative, spatially-explicit projections of the current and future impact of control programs against a disease. These estimates can then be used at a local level to identify the effectiveness of suggested intervention schemes and allow investigation of alternative strategies. The methodology has been applied to lymphatic filariasis in East Africa to provide estimates of the impact of different intervention strategies against the disease.

## Highlights

• Novel methodology for combining geostatistical mapping and transmission modelling.
• Guide the planning of spatial control programmes by identifying affected areas.
• Current intervention strategy will not be sufficient to eliminate LF in most areas.
• Alternative strategies will be required to accelerate LF elimination in East Africa.

## 1 Introduction

Geostatistical modelling is increasingly used in epidemiology to combine surveys from multiple locations into a detailed model of local prevalence or incidence (Hay, Guerra, Gething, Patil, Tatem, Noor, Kabaria, Manh, Elyazar, Brooker, et al., 2009, Moraga, Cano, Baggaley, Gyapong, Njenga, Nikolay, Davies, Rebollo, Pullan, Bockarie, et al., 2015, O’Hanlon, Slater, Cheke, Boatin, Coffeng, Pion, Boussinesq, Zouré, Stolk, Basáñez, 2016, Stensgaard, Vounatsou, Onapa, Simonsen, Pedersen, Rahbek, Kristensen, 2011, Giorgi, Diggle, Snow, Noor, 2018). Maps of disease distribution can be used, for example, to plan the development of national scale control strategies by informing policy makers where intervention efforts should be focused (Slater, Michael, 2013, Tatem, Smith, Gething, Kabaria, Snow, Hay, 2010). Several examples from the literature have shown that spatial heterogeneity is an important epidemiological factor in many diseases (Pullan, Sturrock, Magalhaes, Clements, Brooker, 2012, Sturrock, Gething, Clements, Brooker, 2010, for example). However, predictions of future cases are frequently performed on aggregated data, risking the ecological fallacy (Wakefield and Lyons, 2010).

Over the past decades, mathematical models have also been established as an important tool for evaluating the effect of different control strategies by predicting the progression of the disease (Ferguson, Cummings, Cauchemez, Fraser, Riley, Meeyai, Iamsirithaworn, Burke, 2005, Hollingsworth, 2018, Stolk, Prada, Smith, Kontoroupis, De Vos, Touloupou, Irvine, Brown, Subramanian, Kloek, et al., 2018, Tildesley, Bessell, Keeling, Woolhouse, 2009). However, when mathematical modelling is used to evaluate potential intervention strategies, spatial heterogeneity is also frequently ignored (Heesterbeek et al., 2015). Some notable exceptions are the papers by Gibson (1997), Keeling et al. (2001) and Deardon et al. (2010) who considered a spatial model, where the transmission probabilities depend on distances between individuals. In this paper we develop a novel method for taking the output from a geostatistical model and projecting the epidemic dynamics forward in time at the pixel level, under a range of potential intervention strategies, in a computationally efficient way. An important feature of our approach is the ability to capture several sources of uncertainty.

There are only a limited number of studies linking transmission models and geostatistical maps in a way that can dynamically inform policy at a local level. The African Program for Onchocerciasis Control was one of the first groups to develop and apply this approach (for example Alley et al., 1994, Plaisier, Van Oortmarssen, Remme, Alley, Habbema, 1991). Kriging was used to extrapolate between survey points and then transmission models were used to project the likely impact of intervention programs. These mapped projections had been extremely useful in informing policy planning over many years and have recently been updated (Tekle et al., 2016). The power of this type of approach to inform policy has been illustrated most notably by Bhatt et al. (2015) in the analysis of the key drivers of successes in malaria interventions over the last 15 years. An important challenge, addressed by our approach, is to appropriately estimate and communicate the projections with their uncertainty. In particular, our method addresses and quantifies a broad range of uncertainties, including uncertainty in the spatial variation in prevalence, transmission parameters, demographics, interventions and even model structure, and propagates them into the uncertainty in future predictions.

Our methodology has many parallels with exact versions of Approximate Bayesian Computation (ABC; Beaumont, Zhang, Balding, 2002, Wilkinson, 2013), in which simulations from the model are weighted (or accepted) according to their likelihood of producing the observed data. However, in our framework a likelihood is only available at the survey points, and so instead we weight the simulations by the posterior distribution from a geostatistical model that interpolates between surveys to give a prevalence distribution at each location. To achieve this weighting, we must change the measure of the simulated prevalences from the one induced by the prior on the transmission model parameters, to the posterior distribution from the geostatistical model using the Radon-Nikodym derivative (Billingsley, 1995). However, since this is not available in analytical form, we propose an empirical alternative similar to Goldie and Maller (1999) and references contained within.

The paper is organised as follows. In Section 2 we describe the statistical methodology for combining geo-statistical mapping and transmission modelling, and illustrate its key features with a toy example in Section 3. The proposed method is applied in Section 4 to investigate the impact of intervention programs for lymphatic filariasis in seven countries in Africa. Finally, we conclude with a discussion on limitations of the current method and possible extensions for further research in Section 5.

## 2 Methods

We develop a Bayesian methodology that captures uncertainty from multiple sources and can be readily applied to different transmission models and intervention strategies to give a distribution of projections across space. The starting point for our analysis is the output from a geostatistical model of disease prevalence, capturing the uncertainty in the spatial distribution of infection. A number of recent studies have adopted a predictive framework known as model-based geostatistics (Diggle and Ribeiro, 2007) for the production of prevalence maps, often employing Bayesian inference for spatial prediction and robust characterisation of uncertainty surrounding those predictions. In particular we assume that the output consists of M Monte Carlo samples from the posterior distribution of the geostatistical model. Although we assume that the spatial distribution represents the pre-control prevalence here, our methodology can easily be generalised beyond this example. In addition, we assume that other geographical information is available for each pixel (with associated uncertainty), such as population size and other demographic data that can be used as an input to the transmission model.

**Fig. 1:**
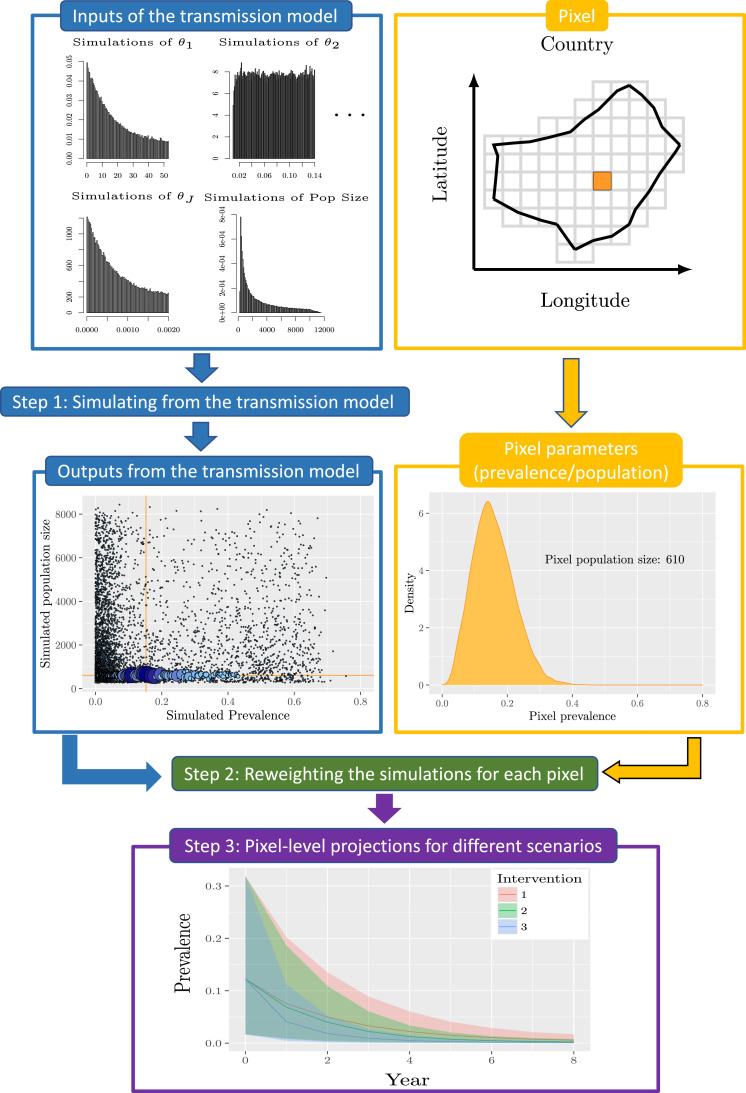
Methodology for generating mapping results. Using pre-run model simulations (top), we reweight the simulations for each pixel based on the prevalence and population information (middle – red lines represent the median of observed data). Finally, the weights are used to evaluate the impact of different intervention strategies (bottom).

Our methodology consists of 3 steps. First, we generate a large number of simulations from the transmission model, with sufficient variability to capture all of the endemic prevalences observed in the samples from the geostatistical model. Second, for each spatial location in the map we reweight the simulations according to how similar they are to the observed prevalence and other spatial information, such as population data. Finally, we simulate the transmission model further forward in time, possibly under some intervention strategy, and apply the weights to obtain the spatial distribution of the projections. A graphical representation of the method can be found in Fig. 1.

### 2.1 Step 1: simulating from the transmission model

For each pixel on the map, we assign an informative prior on the model parameters, $\pi_{i}\left(\theta \right)$ say for pixel i, representing the uncertainty in our beliefs about the parameters of the transmission model at that location. Next, we define a single proposal distribution over the parameter space, q(θ), capable of producing simulated prevalence levels spanning the values observed in the geostatistical mapping. We then draw J parameter vectors ($\theta_{j}$) from the proposal, and for each one we run the model forward in time until it reaches pre-control equilibrium. Denote the resulting prevalence levels by $p_{j},$ for j=1,⋯J. Finally, we calculate an initial I×J matrix of weights for the I pixels and J simulations according to the usual importance sampling formula, namely $w_{\mathrm{ij}}^{\left(1\right)}=\pi_{i}\left(\theta_{j}\right)/q\left(\theta_{j}\right)$.

The proposal distribution q(θ) might be uniform over the parameter space in low dimensions, or for higher dimensions it could be developed from pilot simulations, where parameter vectors are sampled uniformly from the support of the priors and for each parameter vector the equilibrium prevalence is simulated from the transmission model. The importance proposal can then be constructed on the parameter space to give more weight to frequently observed prevalence values, and zero weight to implausible prevalence values (e.g. prevalences larger than the maximum observed in the geostatistical model). The efficiency of the proposal can be improved iteratively using adaptive importance sampling techniques (Cornuet, Marin, Mira, Robert, 2012, Retkute, Touloupou, Basanez, Hollingsworth, Spencer).

### 2.2 Step 2: reweighting the simulations to match pixel prevalence distributions

For each pixel the same simulations are reweighted to match the prevalence distribution of that pixel. This prevents unnecessary replication of simulations for pixels that are broadly similar and means that the number of simulations need not increase as the number of pixels increases. More specifically, for pixel i=1,⋯,I and simulation j=1,⋯,J the new weight is given by:

(1) $$w_{\mathrm{ij}}^{\left(2\right)}\propto \frac{f\left(p_{j}|d_{i}\right)}{g(p_{j}\left|w_{i}^{\left(1\right)}\right)}w_{\mathrm{ij}}^{\left(1\right)}$$

where $d_{i}=\left(d_{i1},\cdots ,d_{\mathrm{iM}}\right)$ is the M dimensional vector of posterior samples of prevalences in pixel i and $w_{i}^{\left(1\right)}=\left(w_{i1}^{\left(1\right)},\cdots ,w_{\mathrm{iJ}}^{\left(1\right)}\right)$. The function f represents the probability of having prevalence $p_{j}$ under the geostatistical model and g represents the probability of simulating prevalence $p_{j}$ from the model with parameter vector drawn from the prior. The ratio f/g therefore represents the usual change of measure formula (Radon-Nikodym derivative). However, since neither of these probability densities are likely to be available in closed form, we use an empirical approximation given by the amount of probability density within δ/2 of $p_{j}$:

$$\begin{array}{cc} f\left(p_{j}|d_{i}\right)= & \frac{1}{\delta M}\sum_{m=1}^{M}1_{\left\{p_{j}-\delta /2\;\leq \;d_{im}\;\leq \;p_{j}+\delta /2\right\}},\\ g(p_{j}\left|w_{i}^{\left(1\right)}\right)= & \frac{\sum_{k=1}^{J}w_{ik}^{\left(1\right)}1_{\left\{p_{j}-\delta /2\;\leq \;p_{k}\;\leq \;p_{j}+\delta /2\right\}}}{\delta \sum_{k=1}^{J}w_{ik}^{\left(1\right)}}. \end{array}$$

Note that as long as q(θ)>0 implies $\pi_{i}\left(\theta \right)>0,$ then $w_{\mathrm{ij}}^{\left(1\right)}>0$ for all j and hence $g(p_{j}|w_{i}^{\left(1\right)})>0$ for all j. The bin width δ controls the trade-off between effective sample size and the fidelity of the distribution of the simulated prevalences to the geostatistical posterior distribution, and should be set as small as possible, whilst producing a reasonable effective sample size, defined as ${\left(\sum_{j=1}^{J}w_{\mathrm{ij}}^{\left(2\right)}\right)}^{2}/\sum_{j=1}^{J}{\left(w_{\mathrm{ij}}^{\left(2\right)}\right)}^{2}$. Finally, the weights from Eq. (1) are normalised to give the posterior probabilities (according to the geostatistical model) that simulation j is appropriate for pixel i.

### 2.3 Step 3: running the simulations forward

The simulations are run forward in time under a given intervention strategy. For each pixel the projected outcomes are weighted according to the normalised weights $w_{i}^{\left(2\right)}=\left(w_{i1}^{\left(2\right)},\cdots ,w_{\mathrm{iJ}}^{\left(2\right)}\right)$ produced in Step 2. Step 3 is repeated for each intervention strategy under consideration.

### 2.4 Lemma on the change of measure

In this section we introduce a lemma that generates the reweighting formula in Eq. (1). The lemma is proved in Appendix A.1 of the Supplementary Material (SM).

Lemma 1 *Let*$p:R^{d}\to \left[0,1\right]$*denote a deterministic model that produces a prevalence*p(θ)*from a vector of parameters*θ*. Let*π(θ)*be a prior distribution over the parameters that induces a prior distribution over prevalences, which we denote by*g(p)*. Suppose that there exists a differentiable and invertible function*$\phi :R^{d}\to R^{d}$*that admits the prevalence as its first argument, ie.*ϕ(θ)=(p(θ),q(θ))*for some*q(θ)*. Finally suppose that we wish to change the probability measure over prevalences from*g*to another measure*f*that is absolutely continuous with respect to*g*. Then the resulting measure over the parameter space is given by*$h\left(\theta \right)=\frac{f(p(\theta ))}{g(p(\theta ))}\pi \left(\theta \right).$

**Notes:**

1. The same approach can be applied for stochastic transmission models as long as the model is defined on a separate probability space (Ω,F,P) to the prior. For stochastic models we fix ω∈Ω and consider the transmission model as a deterministic map ϕ(θ,ω), applying the Lemma and then integrating over Ω.
2. The condition that f must be absolutely continuous with respect to g means that whenever g(p)=0 then we must also have f(p)=0. In other words, when the prior probability of a prevalence is zero then the map measure of prevalence must also be zero. This has important implications for the implementation of our method, discussed further in Appendix A.2.

### 2.5 Alternative empirical Radon-Nikodym derivatives

In Step 2 of our algorithm (described in Section 2.2) we proposed an empirical estimate of the Radon-Nikodym derivative f/g based on using the prevalences within δ/2 of $p_{j}$. Clearly there are many possible alternative estimates that could be used and there are two in particular that are worthy of further discussion. The first is based on histograms and the second is based on minimising a discrepancy measure.

#### 2.5.1 Histogram-based empirical Radon-Nikodym derivative

If we consider a fixed partition of the prevalence space into bins (as if we were constructing a histogram) then it is straightforward to calculate the Radon-Nikodym derivative f/g for each bin as the proportion of posterior samples in the bin divided by the proportion of the weight belonging to simulations that fall in the bin. More precisely, given a finite set of disjoint intervals with union [0,1] then if prevalence $p_{j}$ falls in interval $I(p_{j})$ we have that

$$\begin{array}{ccc} & & f\left(p_{j}|d_{i}\right)=\frac{1}{M\left|I\left(p_{j}\right)\right|}\sum_{m=1}^{M}1_{\left\{d_{im}\in \;I\left(p_{j}\right)\right\}},\\ & & g(p_{j}\left|w_{i}^{\left(1\right)}\right)=\frac{\sum_{k=1}^{J}w_{ik}^{\left(1\right)}1_{\left\{p_{k}\in \;I\left(p_{j}\right)\right\}}}{|I\left(p_{j}\right)|\sum_{k=1}^{J}w_{ik}^{\left(1\right)}}, \end{array}$$

where $\left|I(p_{j})\right|$ is the length of the interval containing $p_{j}$.

The main advantage of this estimate is computational – since all of the simulations in the same interval have the same ratio (for a given pixel) then instead of having to calculate J ratios we need only calculate one per interval. A secondary advantage is that the weighted histogram of the simulation prevalences will be identical to the histogram of the posterior prevalence distribution. However, the relative weightings within each bin are unchanged and so a different choice of bins will reveal that the two distributions are different.

#### 2.5.2 Discrepancy-based empirical Radon-Nikodym derivative

A second alternative empirical Radon-Nikodym derivative can be defined to minimise the difference between the empirical cumulative distribution functions (cdfs) of the posterior prevalences and the weighted simulated prevalences. Let $F\left(x|d_{i}\right)=\frac{1}{M}\sum_{m=1}^{M}1_{\left\{d_{\mathrm{im}}\leq x\right\}}$ be the empirical cdf of the map prevalence distribution for pixel i and $H\left(x|w_{i}^{\left(2\right)}\right)=\sum_{j=1}^{J}w_{\mathrm{ij}}^{\left(2\right)}1_{\left\{p_{j}\leq x\right\}}$ be the empirical cdf of the final weighted distribution of simulated prevalences, then we can choose $w_{i}^{\left(2\right)}$ to minimise some distance $∥F\left(\cdot |d_{i}\right)-H\left(\cdot |w_{i}^{\left(2\right)}\right)∥$. For example, we may wish to minimise $\int_{0}^{1}\left|F\left(x|d_{i}\right)-H\left(x|w_{i}^{\left(2\right)}\right)\right|\;dx$ or $\int_{0}^{1}{\left(F\left(x|d_{i}\right)-H\left(x|w_{i}^{\left(2\right)}\right)\right)}^{2}\;dx$. In this paper we have focussed on the latter of these, for details of the calculation we refer the reader to the SM Appendix A.3.

## 3 Simulation studies: a toy example

In this section we provide a toy example to assess the performance of the proposed method under different settings. Particular focus was given on how the method was affected by the value of δ, by the choice of the proposal distribution of the parameters and the empirical estimate of the Radon-Nikodym derivative. A full description of the analysis can be found in SM Appendix B and here we summarize the key results.

Suppose that the prior distribution is $\pi (\theta_{1},\theta_{2})=2$ if $0<\theta_{2}<\theta_{1}<1$ and zero otherwise. Plots illustrating this prior are given in SM Fig. B.2. For simplicity, assume that the transmission model has equilibrium prevalence given by $p\left(\theta_{1},\theta_{2}\right)=\theta_{1}$ so that the induced prior over prevalences is the marginal for $\theta_{1},$ ie. g(p)=2p for 0<p<1, which is a Beta(2,1) distribution. Further, suppose that we are given M=2000 samples from a pixel with prevalence measure f(p)=2(1−p) for 0<p<1, representing a Beta(1,2) distribution. This challenging example allows us to assess how the methodology performs when there are few simulations with low weights in the area of high posterior probability close to p=0.

**Fig. 2:**
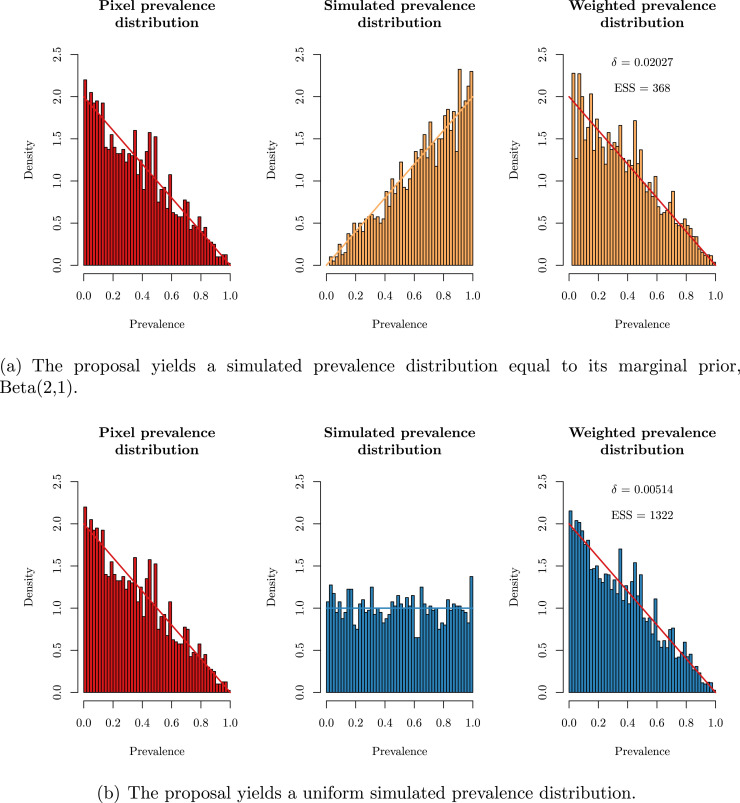
The estimated weighted prevalence distribution for the suggested value of δ (right panel) is compared to the true pixel prevalence distribution (left panel), under different proposal distributions for the prevalence (middle panel): (a) Beta(2,1) and (b) U(0,1). The target densities are also shown on each panel.

Simulations were conducted to investigate the accuracy and efficiency of the proposed method under different settings, where the observed pixel and simulated prevalence data are obtained from the toy model. Fig. 2 shows how J=2000 simulations from a proposal (centre histogram) can be reweighted (right histogram) to resemble the pixel prevalence distribution (left histogram). In Fig. 2(a) the proposal is from the prior, whilst in Fig. 2(b) the proposal is U(0,1). The improvement due to the proposal distribution having good support in all areas of the posterior distribution was demonstrated by the substantial increase in effective sample size (ESS; from 368 to 1322), despite a much smaller value of δ.

**Fig. 3:**
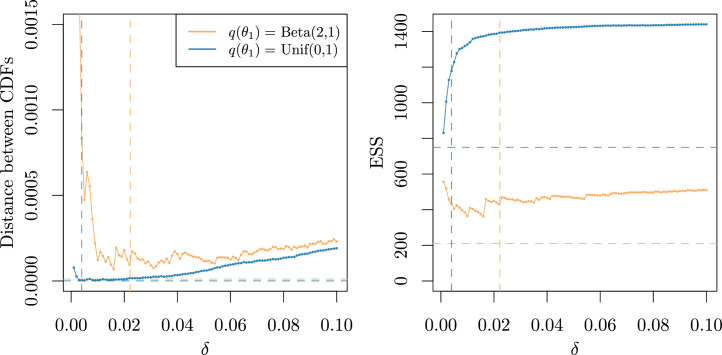
Distance between the two cumulative distribution functions (cdfs) (left panel) and effective sample size (ESS) (right panel) obtained under different values of δ and choice of proposal distribution for parameter $\theta_{1},$ for one randomly selected simulated dataset. Orange solid line represents a prevalence proposal distribution equal to the marginal prior, i.e. Beta(2,1), whereas the blue line corresponds to a U(0,1) proposal. In both cases, the pixel prevalences were drawn from a Beta(1,2) distribution. Dashed vertical lines represent the suggested value of δ for each scenario considered. Dashed horizontal lines correspond to the minimum possible distance (left panel) and its associated ESS (right panel).

Fig. 3 illustrates how the performance changes as δ is increased. The left figure shows the distance (given by integrated squared difference) between the empirical cumulative distribution functions of the weighted simulations and the samples from the pixel posterior; and the right figure shows the effective sample sizes. The corresponding results from the discrepancy based empirical Radon-Nikodym derivative (see Section 2.5.2) are shown as horizontal dashed lines and provide the minimum distance possible between the cdfs. The results show that smaller values of δ reproduced the empirical cdf more accurately, unless δ was so small that very few simulations were included in each estimate of the density g (the denominator in Eq. (1)). After some experimentation (see SM Appendix B.1.1) we chose to set δ to be the smallest value for which at least three simulations were included in each estimate of g. These values are illustrated by vertical lines on Fig. 3, and can be seen to come close to achieving the minimum possible squared distance between the empirical cdfs, but with larger ESS.

In our simulations we have evaluated the performance of the method for the distance-based empirical Radon-Nikodym derivative, which is based on using the prevalences within a certain distance given by δ/2 of $p_{j}$. We also investigated alternative derivatives, discussed in Section 2.5, and the results are summarised in Table B.1 of the SM. Overall, we observed that the discrepancy-based derivative provides the best possible distance between the two cdfs, but at a cost of a lower ESS in all the scenarios considered. When the proposal was uniform and had simulations in all areas of the posterior distribution then the histogram-based derivative performed better than the distance-based derivative both in terms of accuracy and ESS. However, the situation was reversed when there were areas in the proposal with few simulations. In that scenario, the distance-based derivative was found to have lower integrated squared distance and higher ESS compared to the histogram-based derivative. Therefore, we used the distance-based empirical Radon-Nikodym derivative in our applications, since it was more robust to weaknesses in the proposal.

## 4 Application to lymphatic filariasis data

In this section, we apply the proposed approach for the analysis of real data for lymphatic filariasis (LF) in East Africa. LF is caused by a mosquito-borne macro-parasite, which was historically endemic in many tropical countries, with over a billion people at risk of infection, and millions affected by the disease suffer from disability, stigma and associated social and economic consequences (Ramaiah and Ottesen, 2014). LF is one of the neglected tropical diseases (NTDs) targeted for elimination as a public health problem by 2020 (WHO, 2012), with new guidelines currently being developed for 2030. Global efforts to eliminate LF as a public health problem, through the use of mass drug administration (MDA) of treatments with an excellent safety record, have reduced prevalence to low levels in many settings (Ramaiah and Ottesen, 2014). While many countries have successfully scaled-up their programs, there remain a number of questions on how best to scale up treatment to assist priority countries in optimising interventions to accelerate elimination. Therefore, there is an urgent need to provide detailed estimates of the impact of current and future control programs for donor and policy planning.

For LF, the intervention strategy for most of Africa is to have yearly MDA at 65% coverage for 5 years, followed by an assessment of transmission and, if necessary, further rounds of treatment. In areas where MDA has not yet started, alternative strategies may be required to meet the WHO target, i.e. the prevalence being less than 1% (WHO, 2012) as soon as possible. Enhanced strategies include MDA at high coverage or twice-yearly treatment (Stolk et al., 2018). By bringing together statistical mapping and transmission modelling, we aim to provide high-resolution quantification of the likely impact of control programs and predictions on both future impact and demand for interventions, allowing policy makers to more effectively target available resources.

### 4.1 The mathematical model of LF transmission dynamics

In this section we describe the mathematical model of lymphatic filariasis transmission, TRANSFIL (Irvine et al., 2015), that is used throughout the paper. TRANSFIL is an individual-based model of LF infection in human populations, with each host having their own adult worm and microfilariae (mf) burden, as well as mosquito bite risk and treatment history. A full description of the model is provided in Irvine et al. (2015) and in Appendix C of the SM, so here we provide only a brief overview and the updated aspects of it.

Each human is assumed to have their own burden of male and female worms denoted by $W_{i}^{m}$ and $W_{i}^{f},$ respectively. The times at which human i acquires female and male adult worms are given by two inhomogenous Poisson processes, both with rate:

$$\frac{1}{2}\lambda b_{i}\left(V/H\right)\psi_{1}\psi_{2}s_{2}h\left(a\right),$$

where λ is the number of bites per mosquito, V/H is the ratio of vectors to hosts, $\psi_{1}$ is the probability that a third-stage larvae (L3, the infectious stage) leaves the host during a bite, $\psi_{2}$ is the probability that the L3 enters the host, $s_{2}$ is the proportion of L3 that develop into adult worms within the host and h(a) is the biting rate for a human with age a. Both male and female worms are introduced to a human according to a bite risk $b_{i}$ drawn from a gamma distribution with mean 1 and shape parameter k. Thus, the degree of parasite aggregation amongst humans can be quantified by this shape parameter. Finally, we assume that each worm has a constant death rate μ.

Microfilariae concentration in the peripheral blood, denoted by $M_{i},$ is also modelled for each individual according to the following equation:

$$\frac{\text{d}M_{i}}{\text{d}t}=\alpha W_{i}^{f}1_{\left\{W_{i}^{m}>0\right\}}-\gamma M_{i},$$

with α being the production rate of mf per worm, γ the constant death rate of mf and the indicator function $1_{\left\{W_{i}^{m}>0\right\}}$ is one if there are male worms and zero if not. Larvae development occurs when mf enter the mosquito during a blood meal from an infected host. Different functional forms have been found to describe the relationship between the number of mf ingested and the number that develop within the mosquito. For *Anopheles*, which is the genus of the most dominant vector species in East Africa, this relationship is expressed as:

$$L\left(m\right)=\kappa_{s2}{\left(1-e^{-r_{2}m/\kappa_{s2}}\right)}^{2},$$

where m is the concentration of mf per 20 µL taken during a blood meal and $r,\kappa_{s}$ denote the saturation values related to the uptake function as detailed in Gambhir and Michael (2008). The equilibrium value for L3 in a mosquito is given by:

$$L^{*}=\frac{\lambda g\tilde{L}}{\sigma +\lambda \psi_{1}},$$

where λ is the number of bites per mosquito, g is the proportion of mosquitoes which pick up infection when biting an infected host, σ is the death rate of mosquitoes and $\tilde{L}$ is the average number of larvae per mosquito.

Each human begins life with zero infection and a bite-rate of exposure $b_{i}$. The human death rate is denoted by τ and is assumed to be constant throughout an individuals lifetime with a cut-off at age 100. When an individual dies another one is born in order to keep the population size constant.

During an intervention campaign, the impact of MDA is simulated for an individual by reducing their mf concentration and their male and female worm burden according to the estimated drug efficacies from the literature (Ismail, Jayakody, Weil, Nirmalan, Jayasinghe, Abeyewickrema, Sheriff, Rajaratnam, Amarasekera, De Silva, et al., 1998, Michael, Malecela-Lazaro, Simonsen, Pedersen, Barker, Kumar, Kazura, 2004). In addition, there is a period after MDA during which the production of mf for that individual is diminished. Furthermore, the individuals’ compliance after multiple rounds of treatment is modelled based on the paper by Griffin et al. (2010), where the authors model the probability of an individual making the same decision as in the previous round of treatment.

Finally, we extended the model to include a very low rate of importation of infection from outside the population being modelled, otherwise the equilibrium distribution (steady state), that is used as the starting point of the simulations, is just the degenerate distribution where no-one is infected. The interventions reduce the prevalence over time, and so we reduce the importation rate after intervention in proportion to the reduction in prevalence seen in pilot simulations. Lists of the model parameters are provided in Tables C.3 and C.4 of the SM.

### 4.2 Implementation details

**Fig. 4:**
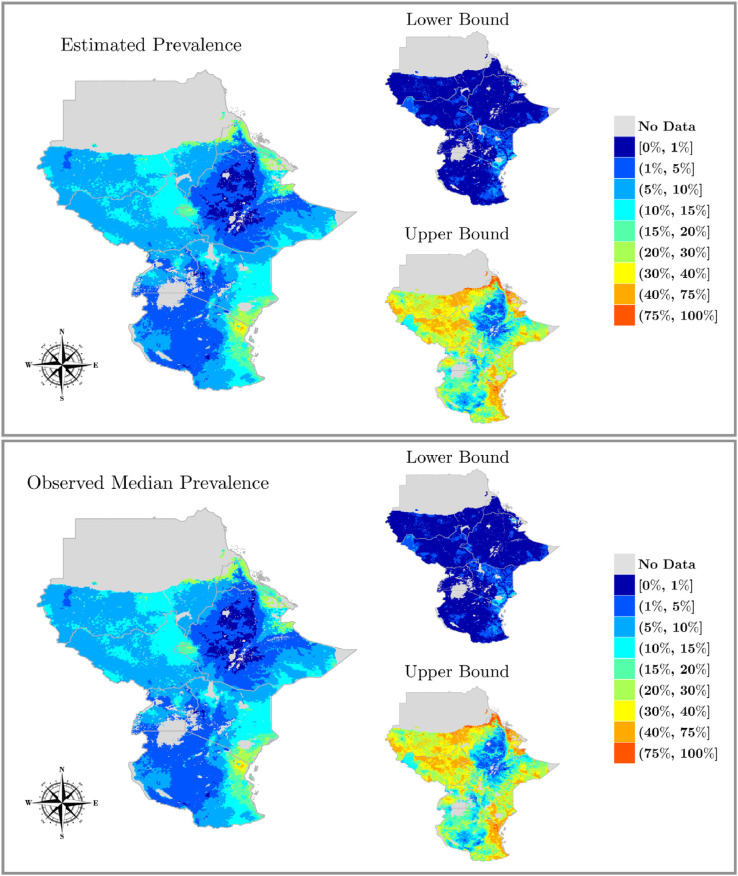
Accuracy assessment of our method. The true distribution of LF prevalence (lower panel) is compared to the estimated prevalence distribution (upper panel) using our proposed methodology predicted at 5×5 km resolution. Point estimates along with lower (2.5%) and upper (97.5%) percentiles are presented.

The starting point for our analysis was the spatial map (pixel scale 5×5 km) providing the predicted distribution of the LF prevalence based on mf data, generated through a Bayesian geostatistical modelling approach described by Moraga et al. (2015). The top panel of Fig. 4 shows the median of the posterior distribution of the prevalence obtained at each pixel, along with estimates of lower (2.5%) and upper (97.5%) percentiles. In particular, we analysed the following seven African countries: Ethiopia, Sudan, South Sudan, Eritrea, Kenya, Tanzania and Uganda. We linked each pixel to the corresponding population estimates obtained from the Gridded Population of the World (Worldpop, 2010), which provides the estimated number of people in each pixel. We avoided handling pixels with either very small or very large populations as the transmission model was not thought to be appropriate in these environments (Irvine, Reimer, Njenga, Gunawardena, Kelly-Hope, Bockarie, Hollingsworth, 2015, Smith, Singh, Irvine, Stolk, Subramanian, Hollingsworth, Michael, 2017). More specifically, for small populations we pooled pixels with less than 300 people together, ensuring that the merged pixels belong to the same country and that the groups contain as few pixels as possible. We excluded pixels with population estimates over 10 000 from the analysis; resulting in 1.7% of the pixels being excluded.

The stochastic model of LF transmission TRANSFIL was used to investigate and compare the impact of different control strategies. In order to simulate the entire range of observed baseline mf prevalence levels, with values up to 95%, we assumed that four parameters of the mathematical model were spatially varying: the population size, the vector to host ratio, the aggregation parameter of individual exposure to mosquitoes and the importation rate, using prior distributions informed from data, pilot simulations and previous analyses. We assumed that the parameter prior was the same in each spatial location (discussed in more details in Appendix C.2 of the SM) except for the population size, which was assumed to be a log normal distribution, ie $\log\left(n\right)∼N(\log\left(N_{i}\right),\sigma^{2}),$ where $N_{i}$ is the reported population of pixel i (adjusted population from Worldpop, 2010) and σ is the sample standard deviation of the log population estimates available in WorldPop.

The proposal density of the population sizes, q(n), was designed so that each simulation contributed an equal amount to the effective sample size of a set of pixels with populations {260,261,⋯,10000}. This was achieved by calculating the effective sample size of an initial proposal, namely, $q_{0}\left(n\right)\propto 1$. The remaining population sizes (10001–11550) were taken to decrease linearly from q(10000) to zero. Since the uncertainty in the log-normal prior is much greater for large populations, fewer simulations are needed in these regions. The final proposal was obtained from 10 iterations of $q_{i}\left(n\right)\propto q_{i-1}\left(n\right)/{\text{ESS}}_{i-1}\left(n\right),$ where ${\text{ESS}}_{i}\left(n\right)$ is the effective sample size of a simulation with n individuals from the proposal $q_{i}$ (see Fig. C.7(a) of the SM).

A significant merit of our approach is that it can be easily applied in parallel which can be utilised to speed up implementation, especially in applications involving a large number of pixels. This is because we are treating each pixel independently and therefore the computation of the weights can be undertaken in parallel. In our application, the computation time of this step was approximately 8 hours using a 112 core computer cluster (around 30 s on a single core for each pixel).

### 4.3 Results

In this section, the Bayesian approach presented in Section 2 was applied to the LF data. Firstly, we assessed the accuracy of the method, defined as the ability of the transmission model weighted simulations to reproduce the pre-control (baseline) geostatistical map, by comparing the observed and the estimated distribution of the baseline (equilibrium) mf prevalences at each pixel. Fig. 4 illustrates the median map (with 2.5 and 97.5 percentiles), along with the corresponding maps of the observed data. Overall, the results show that the maps are almost identical, indicating that the method is able to reproduce the distribution of the observed baseline prevalence in each pixel. In addition, in the left panel of Fig. D.8 of the SM we compared the estimated number of people per pixel with the observed value, which were in close agreement indicating that the proposed method accurately reproduced the number of people in each pixel. In the right panel of Fig. D.8 of the SM, we examined the ESS per pixel, which represents the effective number of simulations per pixel and is a measure of how well the method performs. We observed that the pixels with high prevalence (which may require a change of intervention strategy) have high ESS.

**Fig. 5:**
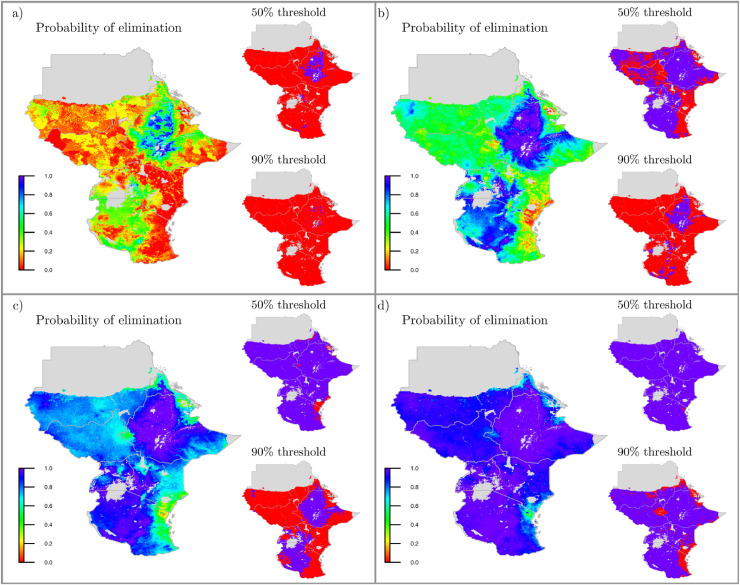
Probability of less than 1% prevalence after 5 years under: a) no intervention; annual MDA with coverage of b) 65%; c) 80% and d) biannual MDA at 65% coverage predicted at 5×5 km resolution, for Ethiopia, Sudan, South Sudan, Eritrea, Kenya, Tanzania and Uganda. Right of panels: Pixels that achieve elimination (blue) or do not achieve elimination (red) using different probability thresholds.

**Fig. 6:**
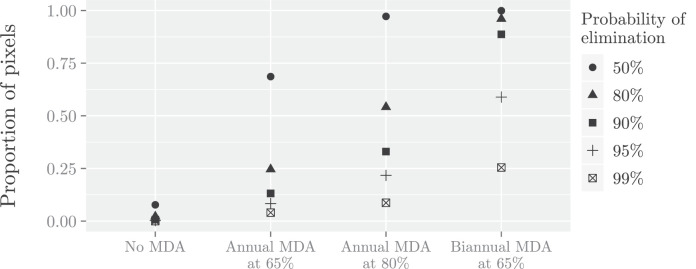
Proportion of pixels with prevalence less than 1% using different probability thresholds after 5 years under four intervention strategies.

Secondly, the methodology was applied to evaluate the impact of different intervention programs for LF in East Africa. In particular, four treatment scenarios were simulated: no interventions; the standard 65% coverage annual MDA (aMDA); 80% coverage aMDA; or biannual MDA (bMDA) at 65% coverage, in order to investigate how these affect the probability of elimination after 5 years (Fig. 5). Adopting a prevalence of less than 1% as the threshold set by WHO as a global target for determining LF transmission elimination, our analysis predicted that the recommended strategy of 5 rounds of aMDA at 65% is not enough for eliminating the disease in all pixels, with probability of elimination above 90% only for 13% of the pixels (see also Fig. 6). Moreover, when more intensive treatments were implemented, i.e. more frequent MDA or higher coverage, the probability of elimination significantly increased compared to aMDA programme at 65%. In particular, bMDA at 65% coverage was the most effective of all strategies considered and was able to achieve elimination in 88% of the pixels, with at least 90% probability. However, the proportion of pixels which achieved elimination after 5 years reduced to 59% and 25% when the probability threshold was increased to 95% and 99%, respectively, illustrating that the policy is sensitive to uncertainty.

Finally, predictions of mf prevalence for the first and fifth year of intervention were summarized by calculating the estimated prevalence at each pixel, together with the 2.5th and 97.5th percentiles in Figs. D.9 and D.10 of the SM, for each of the four scenarios. Very similar observations were made on the predictions of mf prevalence for the first 5 years of intervention.

## 5 Discussion

This study highlights the value of integrating geostatistical prevalence maps and transmission models for providing predictions on the impact of interventions aiming to eliminate transmission at a local scale. The main contribution is the development of new statistical tools through which existing research in mapping and predictive modelling are combined in a computationally efficient and flexible way which correctly accounts for uncertainty in these different techniques. Although we focus and apply our methodology on LF transmission, it can be applied to other infectious diseases.

We have shown that the current strategy of 5 annual rounds of MDA at 65% coverage will not be sufficient to eliminate the disease in most areas. We also found that a change in the current MDA strategy, such as increasing the coverage and frequency of MDA, will be required if LF elimination is to be accelerated in East Africa. This suggests that it may be necessary to employ different enhanced intervention plans at a fine scale, according to the characteristics of each area, in order to achieve the WHO elimination targets.

However, for the results presented here we assumed that no interventions have been applied in East Africa prior to the prevalence survey. While this assumption is correct for most areas, MDA programs began to be implemented in a few districts of Africa since 2000 and in many more districts thereafter. Therefore, one of the next steps will be to account for previous MDA programmes as spatio-temporal covariate information in the transmission model. Apart from MDA, insecticide treated bednets have been used in some countries (data can be extracted from the Malaria Atlas Project), the use of which has been shown to be an effective additional measure for control of the disease (Bockarie et al., 2009). Integrating geostatistical maps with transmission models with these additional covariates is more complicated as the simulations must include the appropriate historical interventions.

An additional challenge is the gap between reported and true coverage with an MDA. Where there are parasitological data against which to test the expected and achieved impact of reported coverages, they have been shown to be unreliable (Budge et al., 2016). This will pose a particular challenge to interpreting historic coverage and a challenge in communicating future projections. This work represents our initial framework and future research will be required to extend the methodology to capture these more complex settings.

A limitation of our statistical approach is that it doesn’t capture the spatial correlations in the predictions, since each pixel is weighted independently to produce a marginal posterior for each pixel. This approach means that we lose the spatial autocorrelation that was captured in the original geostatistical model and, furthermore, that there is no way for nearby pixels to interact during the simulations, for example, to account for movements of humans or vectors. A more sophisticated approach would be to use the spatial autocorrelations from the geostatistical model, alongside any available movement or connectivity data, to define a transmission kernel that describes spatial spread. This kernel could be used within a single meta-population model describing the transmission dynamics across the whole map. At present, such an approach would be computationally infeasible at the country scale, but may become possible in future through improvements in methodology and advances in high-performance computing.

## Appendix A Supplementary materials

Supplementary Data S1 Supplementary Raw Research Data. This is open data under the CC BY license http://creativecommons.org/licenses/by/4.0/ Supplementary Data S1
