# Supplementary material for: Statistical methods for linking geostatistical maps and transmission models: Application to lymphatic filariasis in East Africa
Source: Spat Spatiotemporal Epidemiol. 2022 Jun;41:None. doi: 10.1016/j.sste.2020.100391 (PMC9205338; doi:10.1016/j.sste.2020.100391)
Supplement: Supplementary Data S1 — Supplementary Raw Research Data. This is open data under the CC BY license http://creativecommons.org/licenses/by/4.0/ [file mmc1.pdf]

Supplementary Material to "Statistical methods for linking geostatistical maps and transmission models: Application to lymphatic filariasis in East Africa"

Appendix A. Background information on the methodology

Appendix A.1. Proof of lemma on the change of measure

Proof. First we need to change the variables of the prior from $\boldsymbol{\theta}$ to the transformed variables $(p, \boldsymbol{q})$. This results in a transformed prior density $\pi'(p, \boldsymbol{q}) = \pi(\boldsymbol{\phi}^{-1}(p, \boldsymbol{q}))|J^{-1}|$, where J^{-1} is the determinant of the Jacobian matrix of partial derivatives of $\boldsymbol{\phi}^{-1}$. Next, we wish to apply the Radon-Nikodym derivative formula to change the measure over prevalences from $g(p)$ to $f(p)$, whilst keeping the measure over $\boldsymbol{q}$ unchanged. More formally, we rewrite $\pi'(p, \boldsymbol{q}) = \pi'(p)\pi'(p|\boldsymbol{q}) = g(p)\pi'(p|\boldsymbol{q})$ and change the whole measure from $g(p)\pi'(p|\boldsymbol{q})$ to $f(p)\pi'(p|\boldsymbol{q})$. This yields the new measure in terms of the transformed variables $h'(p, \boldsymbol{q}) = \frac{f(p)\pi'(p|\boldsymbol{q})}{g(p)\pi'(p|\boldsymbol{q})}\pi'(p, \boldsymbol{q}) = \frac{f(p)}{g(p)}\pi'(p, \boldsymbol{q})$. The final step is to transform back to the original variables $\boldsymbol{\theta}$.

$$
\begin{aligned}
h(\boldsymbol{\theta}) &= h'(\phi(\boldsymbol{\theta}))|J| \\
&= \frac{f(p(\boldsymbol{\theta}))}{g(p(\boldsymbol{\theta}))}\pi'(\phi(\boldsymbol{\theta}))|J| \\
&= \frac{f(p(\boldsymbol{\theta}))}{g(p(\boldsymbol{\theta}))}\pi(\boldsymbol{\theta}).
\end{aligned}
$$

□

Appendix A.2. Implementation considerations from the absolute continuity condition

The absolute continuity condition in Lemma 1, that f must be absolutely continuous with respect to g, means that whenever $g(p) = 0$ then we must also have $f(p) = 0$. In other words, when the prior probability of a prevalence is zero then the map measure of prevalence must also be zero. This has important considerations for implementing our method. For example, we find that our prior for the transmission model parameters is unlikely to produce any simulations with a prevalence of above 85%. However, prevalences this high do appear in the tails of the posterior distribution in the geostatistical model, especially when the amount of uncertainty is high. When the weighting is applied, there are no simulations to capture the high prevalence region, which leads to an underestimation of the mean prevalence, for example.

There are several potential approaches to ameliorate this. The simplest would be to mask out areas of the map where this occurs. However, there may be large areas of the map that have only a very small probability of producing a prevalence above 85% according to the geostatistical model. A second approach, which we could take if we believed that prevalences above 85% were a priori impossible, would be to remove any samples from the geostatistical map that went above 85%. This is effectively the same as applying the weighting naively, and leads to the mean and median of the prevalence distribution of the simulations being lower than the corresponding statistics from the geostatistical model. A final more sophisticated approach could be to shift the weight from high prevalences in the geostatistical map to the nearest prevalences available in the simulations. For example, by using

the histogram-based empirical Radon-Nikodym derivative with a wide bin capturing all prevalences above 75%. Although this reduces the size of the underestimation, it substantially reduces the effective sample size, as a small number of simulations get very high weight.

Appendix A.3. Minimum discrepancy-based empirical Radon-Nikodym derivative

For each pixel, we would like to minimise the following distance between the empirical cumulative distribution functions (cdfs) of the posterior prevalences and the weighted simulated prevalences:

$$\int_0^1 \left(F(x|\boldsymbol{d}) - H(x|\boldsymbol{w}^{(2)})\right)^2 \, \mathrm{d}x,$$

with respect to the weights $\boldsymbol{w}^{(2)}$. First, the posterior and simulated prevalence samples are sorted in ascending order, such that $d_{(1)} \leq d_{(2)} \leq \ldots \leq d_{(M-1)} \leq d_{(M)}$ and $p_{(1)} \leq p_{(2)} \leq \ldots \leq p_{(J-1)} \leq p_{(J)}$. Similarly, we introduce the notation $w^{(2)}_{(j)}$ for the weights of simulation j which produces prevalence $p_{(j)}$, for $j = 1, 2, \ldots, J$. For clarity of notation, an example of two empirical cdfs is shown in the left panel of Figure A.1.

Starting with $j = 1$, we have that:

$$\begin{aligned}\frac{\partial}{\partial w^{(2)}_{(1)}} \int_{p_{(1)}}^{p_{(2)}} \left(F(x|\boldsymbol{d}) - H(x|\boldsymbol{w}^{(2)})\right)^2 \, \mathrm{d}x &= \frac{\partial}{\partial w^{(2)}_{(1)}} \int_{p_{(1)}}^{p_{(2)}} \left(F(x|\boldsymbol{d}) - w^{(2)}_{(1)}\right)^2 \, \mathrm{d}x \\ &= -2 \left[\int_{p_{(1)}}^{p_{(2)}} \left(F(x|\boldsymbol{d}) - w^{(2)}_{(1)}\right) \, \mathrm{d}x\right] \\ &= -2 \left[\int_{p_{(1)}}^{p_{(2)}} F(x|\boldsymbol{d}) \, \mathrm{d}x - w^{(2)}_{(1)} \left(p_{(2)} - p_{(1)}\right)\right]. \qquad \text{(A.1)}\end{aligned}$$

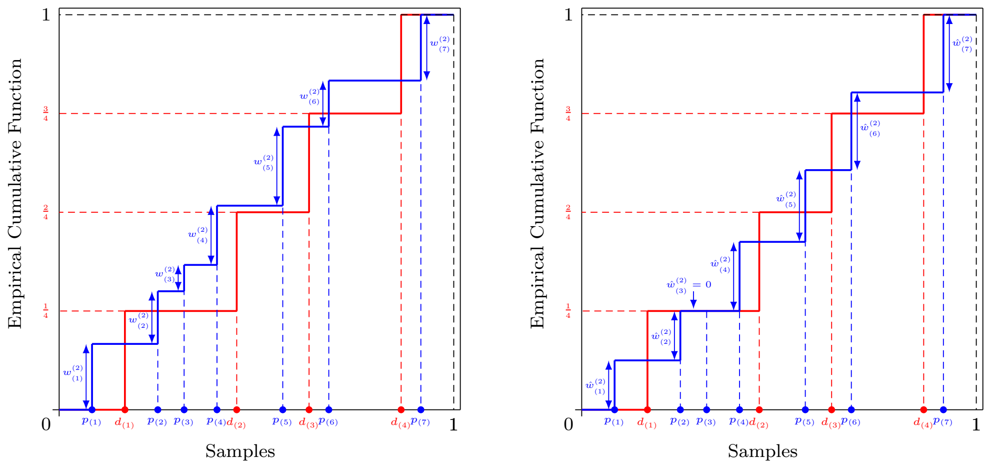

Figure A.1: An example of the empirical cumulative distribution functions of the posterior prevalences (red) and the weighted simulated prevalences (blue). The empirical cdf of the weighted simulated prevalence is shown in the left panel with arbitrary weights and in the right panel using the optimal weights that achieve the minimum distance between the two cdfs, as described in Appendix A.3.

At the minimum Equation (A.1) is equal to 0, therefore:

$$\hat{w}_{(1)}^{(2)} = \frac{\displaystyle\int_{p_{(1)}}^{p_{(2)}} F(x|\boldsymbol{d}) \, \mathrm{d}x}{p_{(2)} - p_{(1)}}.$$

Similarly, we differentiate the distance between the two cdfs with respect to $w_{(j)}^{(2)}$, for $j = 2, 3, \ldots, J-1$ and by setting it equal to 0,

$$\frac{\partial}{\partial w_{(j)}^{(2)}} \int_{p_{(j)}}^{p_{(j+1)}} \left(F(x|\boldsymbol{d}) - \sum_{k=1}^{j-1} \hat{w}_{(k)}^{(2)} - w_{(j)}^{(2)} \right)^2 \mathrm{d}x$$

$$= -2 \left[\int_{p_{(j)}}^{p_{(j+1)}} F(x|\boldsymbol{d}) \, \mathrm{d}x - \left(p_{(j+1)} - p_{(j)}\right) \left(\sum_{k=1}^{j-1} \hat{w}_{(k)}^{(2)} + w_{(j)}^{(2)} \right) \right] = 0,$$

we obtain:

$$\hat{w}^{(2)}_{(j)} = \frac{\displaystyle\int_{p_{(j)}}^{p_{(j+1)}} F(x|\boldsymbol{d})\,\mathrm{d}x}{p_{(J+1)} - p_{(J)}} - \sum_{k=1}^{j-1} \hat{w}^{(2)}_{(k)}.$$

Finally, to ensure that the weights sum to one, for $j = J$ we have that:

$$\hat{w}^{(2)}_{(J)} = 1 - \sum_{k=1}^{J-1} \hat{w}^{(2)}_{(k)}$$

As an example, in the right panel of Figure A.1 we provide the optimal weights $\hat{w}^{(2)}_{(j)}$, i.e. the ones with the minimum distance between the two empirical cdfs.

Appendix B. Toy example

Suppose that the prior distribution is $\pi(\theta_1, \theta_2) = 2$ if $0 < \theta_2 < \theta_1 < 1$ and zero otherwise. The prior support and marginal densities are shown in Figure B.2. For simplicity, assume that the transmission model has equilibrium prevalence given by $p(\theta_1, \theta_2) = \theta_1$ so that the induced prior over prevalences is the marginal for θ_1, ie. $g(p) = 2p$ for $0 < p < 1$. Further, suppose that we are given a pixel with prevalence measure $f(p) = 2(1 - p)$ for $0 < p < 1$. This challenging example allows us to assess how the methodology performs when there are few simulations with low weights in the area of high posterior probability close to $p = 0$.

By the Lemma 1 of the main text, the new measure over the parameter

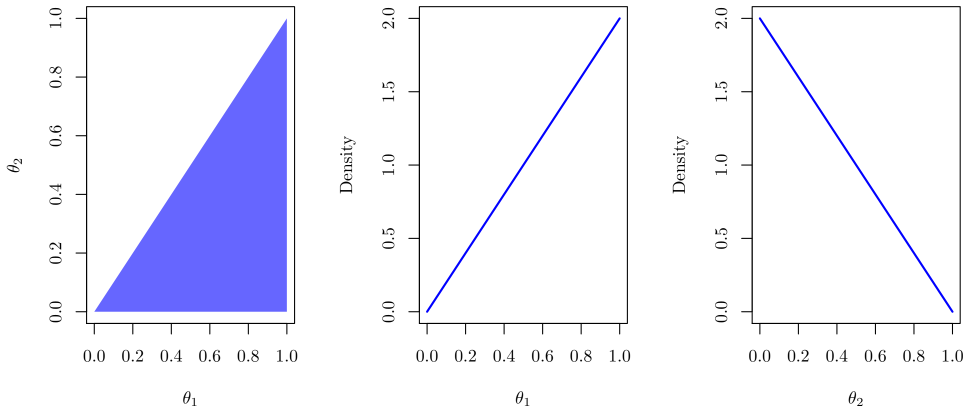

Figure B.2: Prior support and marginal densities for the parameters of the toy example in Appendix B.

space is given by

$$\begin{aligned} h(\theta_1, \theta_2) &= \frac{f(p(\theta_1, \theta_2))}{g(p(\theta_1, \theta_2))} \pi(\theta_1, \theta_2) \\ &= \frac{f(\theta_1)}{g(\theta_1)} \times 2 \\ &= \frac{2(1-\theta_1)}{2\theta_1} \times 2 \\ &= \frac{2(1-\theta_1)}{\theta_1} \qquad \text{for } 0 < \theta_2 < \theta_1 < 1. \end{aligned}$$

First, we can verify that we get the correct marginal for $p = \theta_1$.

$$\begin{aligned} f(p) &= \int h(p, \theta_2)\, \mathrm{d}\theta_2 \\ &= \int_0^p 2\frac{(1-p)}{p}\, \mathrm{d}\theta_2 \\ &= \left[\theta_2 \frac{2(1-p)}{p}\right]_{\theta_2 = p} \\ &= 2(1-p) \qquad \text{for } 0 < p < 1, \end{aligned}$$

as required.

Second, we can determine the new marginal density for θ_2.

$$\begin{aligned} h(\theta_2) &= \int h(\theta_1, \theta_2)\, \mathrm{d}\theta_1 \\ &= \int_{\theta_2}^{1} \frac{2(1-\theta_1)}{\theta_1}\, \mathrm{d}\theta_1 \\ &= 2\left[\log(\theta_1) - \theta_1\right]_{\theta_1=\theta_2}^{1} \\ &= 2(\theta_2 - \log(\theta_2) - 1) \qquad \text{for } 0 < \theta_2 < 1. \end{aligned} \tag{B.1}$$

Appendix B.1. Simulations

In this section, we perform a series of simulations to assess the accuracy and efficiency of the proposed method for recovering the distribution of pixel prevalence. Particular focus is given on how the method is affected as we vary the value of δ, the proposal distribution $q(\boldsymbol{\theta})$, and the empirical estimate of the Radon-Nikodym derivative.

Appendix B.1.1. Sensitivity analysis: Value of δ.

We first investigated the performance of the proposed method as a function of δ, where the observed pixel and simulated prevalence data are obtained from the toy model described in Appendix B. In particular, we generated $M = 2\,000$ pixel prevalence samples from Beta(1,2). We then generated $J = 2\,000$ samples from the joint prior distribution of parameters θ_1 and θ_2; first we drew $\theta_1 \sim$ Beta(2,1) and then $\theta_2 \mid \theta_1 \sim \theta_1 \times$Beta(1,1). Therefore, the obtained simulated prevalence samples are draws from Beta(2,1), which is the marginal prior for θ_1. We considered values for δ from 0.001 through to 0.1, increasing by 0.001 each time.

Accuracy was assessed by computing the KolmogorovSmirnov (KS) distance which is defined as the largest vertical distance between the two empirical cumulative distribution functions of the pixel prevalence and the weighted simulated prevalence. We also consider an additional measure of quantifying the distance between the two cdfs, termed as integrated squared distance and given by $\int_0^1 \left(F(x|\boldsymbol{d}) - H(x|\boldsymbol{w}^{(2)})\right)^2 \, \mathrm{d}x$. The calculation of the two distances is repeated 100 times for each value of δ, using new pixel and simulated prevalence samples each time, in order to prevent biases occurring due to the simulating procedure. Results are shown in Figure B.3. We see that the performance of our method is affected by the value of δ. In the left panel of Figure B.3 we show the median KS distance along with the 95% credible interval, over the 100 realisations for each δ. Overall, the accuracy of the algorithm increases as δ grows from 0.001 to 0.033, where the median KS distance reaches its minimum value, and for δ higher than 0.033 a slight decrease is observed. The integrated squared distance, shown in the middle panel of Figure B.3, provides identical conclusions. Therefore, from now on we use the integrated squared difference to assess the accuracy of the method. An opposite pattern is observed in the efficiency of the method, as can be seen in the right panel of Figure B.3 where we show the effective sample size (ESS) as a function of δ. Note that despite having the highest ESS for $\delta = 0.001$, the method has the lowest accuracy as indicated by the highest distance between the two empirical distributions. This could be attributed to the fact that there are very few simulations in each estimate of density g (the denominator in Equation 1).

This motivated us to propose an appropriate value of δ depending on the

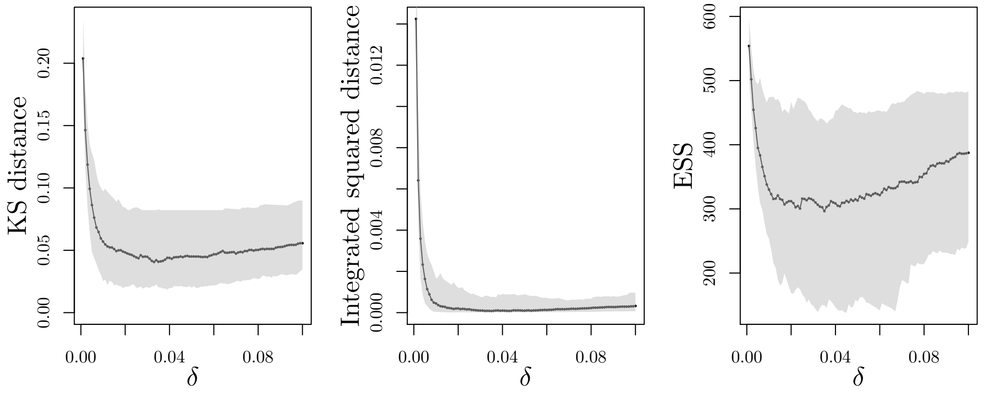

Figure B.3: Sensitivity to δ in the example where the parameters are drawn from their prior distribution. The left panel shows the Kolmogorov-Smirnov (KS), the middle panel the integrated squared distance and the right panel the ESS for $\delta = 0.001, 0.002, \ldots, 0.1$. Shaded areas correspond to the 95% credible interval.

simulated prevalence samples. More specifically, let $\delta_{k,j} = 2 \mid p_k - p_j \mid$, for $j, k = 1, 2, \ldots, J$. For each k, let $\delta_{k,(j)}$ to denote the sorted values with respect to j, such that $\delta_{k,(1)} \leq \delta_{k,(2)} \leq \ldots \leq \delta_{k,(J)}$. Note that $\delta_{k,(1)}$ is always equal to zero since for $k = j$, $\delta_{k,k} = 0$. Finally, the suggested value of δ, denoted by $\tilde{\delta}$, is given by $\tilde{\delta} = \max\left(\delta_{1,(3)}, \delta_{2,(3)}, \ldots, \delta_{J,(3)}\right)$, ensuring that at least 3 simulations were included in each estimate of g. In Figure B.4, we report the median $\tilde{\delta}$ (orange vertical line) along with the 95% credible interval, as obtained from the 100 simulations. Overall, the suggested value of δ achieves a good balance between efficiency and accuracy. In addition, the estimated weighted prevalence distribution of the suggested value of δ is provided in the right panel of Figure 2(a) in the main text and shows that the method correctly reproduces the histogram of the true pixel prevalence (left panel), except maybe for prevalence values close to 0 where the histogram is a bit

noisy. This is justified by the fact that there are only few simulations that have prevalence values between 0 and 0.02, as illustrated in the middle panel.

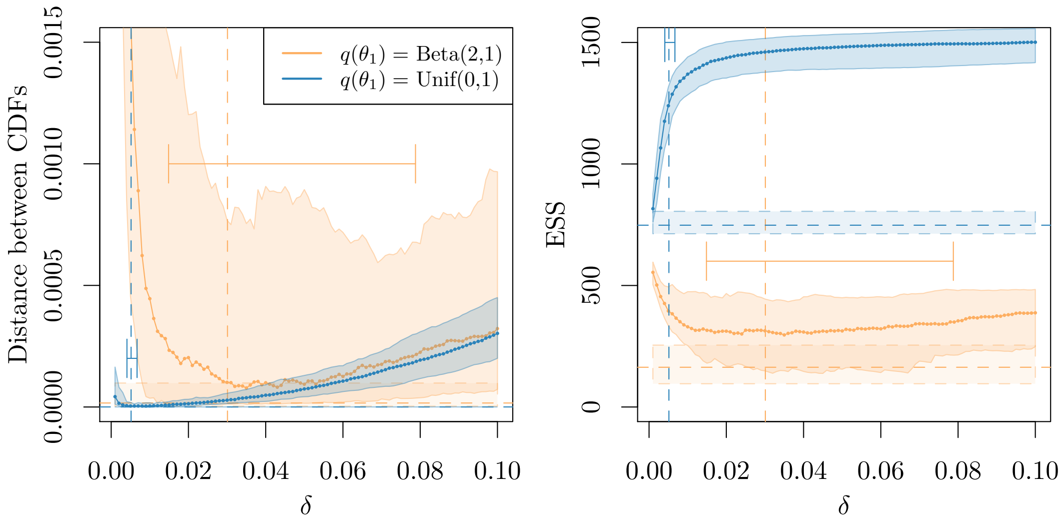

Figure B.4: Integrated squared distance between the two cumulative distribution functions (cdfs) (left panel) and effective sample size (ESS) (right panel) obtained from 100 simulated datasets, under different values of δ and choice of proposal distribution for parameter θ_1. Orange solid line represents a prevalence proposal distribution equal to marginal prior, i.e. Beta(2,1), whereas blue line corresponds to a $U(0,1)$ proposal. In both cases, the pixel prevalences were drawn from a Beta(1,2) distribution. Dashed horizontal lines correspond to the minimum possible distance (left panel) and its associated ESS (right panel). Shaded areas correspond to the 95% credible intervals for each estimate. Dashed vertical lines represent the suggested value of δ for each scenario of the proposal distribution considered, along with their upper and lower limits (error bars).

Appendix B.1.2. Sensitivity analysis: Proposal distribution.

In this section, we carry out a sensitivity analysis to assess the effect that the proposal distribution of the parameters has on the performance of the method. More specifically, instead of drawing θ_1 from its prior distribution

Beta(2,1), we consider a uniform proposal $U(0,1)$. This change results to a uniform distribution over the simulated prevalences. As before, we assess the performance of the method for different values of δ, shown in Figure B.4. Overall, we conclude that the performance of the method improves substantially when we move from the prior to the uniform proposal distribution over the prevalences. This is because there are no areas with strong posterior probability that have few simulations from the proposal. In particular, the median integrated squared distance between the two cdfs (left panel) appears to be much lower in the latter compared to the former, and it is also associated with lower variability of the estimate. In addition, using a uniform proposal distribution leads to substantial increase in ESS (right panel), with the median ESS being at least 1.5 times higher for values of δ close to 0.001 and up to 3.5 times for larger values of δ. For reference, we also display the minimum discrepancy-based empirical Radon-Nikodym derivative, as described in Appendix A.3, and its associated ESS. Overall, we come close to reach the minimum possible integrated squared distance between the two cdfs, with larger ESS.

To further assess the accuracy of the method we compared the weighted posterior distribution of θ_2 with its true density, calculated in Equation (B.1). Results are shown in Figure B.5 for each choice of the proposal distribution. The simulation analysis illustrates that both proposals perform well in terms of recovering the target density of θ_2. Nevertheless, using a uniform proposal for the prevalence leads to a large improvement in the estimate.

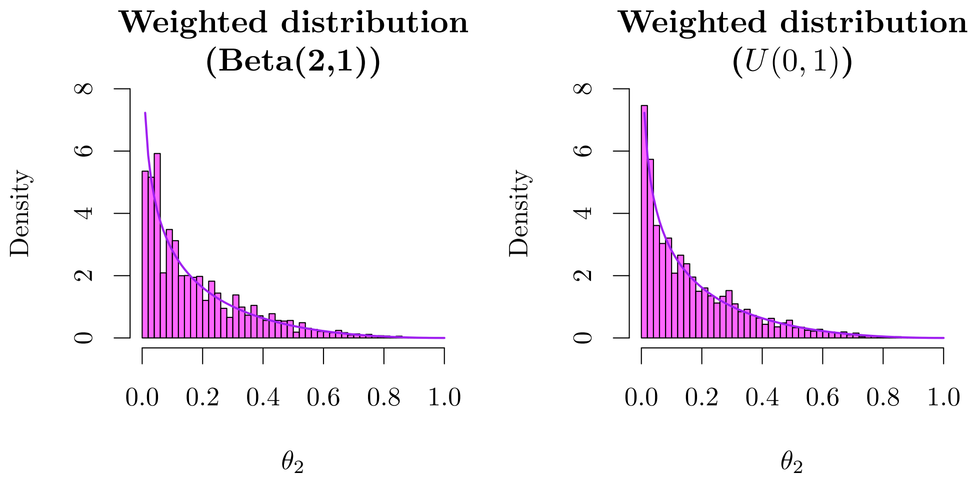

Figure B.5: The estimated weighted posterior distribution of θ_2 is compared to the target density (purple line), under different proposal distributions for the prevalence: Beta(2,1) (left panel) and $U(0,1)$ (right panel).

Appendix B.1.3. Sensitivity analysis: Empirical estimate of the Radon-Nikodym derivative.

In order to study the influence of the empirical Radon-Nikodym derivative (ERND) on the method performance, we repeated our simulation analysis for three different choices: (a) the proposed empirical estimate described in Section 2.2 referred as the distance-based derivative, (b) the histogram-based derivative in Section 2.5.1 and (c) the discrepancy-based derivative using the integrated squared distance (for more details see Section 2.5.2 of the manuscript). Results are given in Table B.1. Note that the proposed distance-based derivative is based on using the prevalences within $\tilde{\delta}/2$ of p_j and the histogram-based derivative is calculated by splitting the $[0,1]$ interval into 100 equal bins.

As expected, the discrepancy-based ERND is optimal in terms of the

distance between the two cdfs, but results in much lower ESS compared to the alternative derivatives. When there are few simulations of the proposal in the area of high posterior probability close to zero prevalence, the distance-based ERND performs better compared to the histogram-based ERND in terms of both integrated squared distance and ESS. When we move to a uniform simulated prevalence distribution, we find that histogram-based derivative outperforms the distance-based derivative, and is the one that scores highest in terms of ESS, followed in order by distance-based and discrepancy-based derivative. However, its performance depends on the choice of bins, since the relative weightings within each bin are unchanged. For example, Figure B.6 uses just 10 bins to illustrate that the distribution within each bin does not reflect the target distribution. Finally, we conclude that the performance of all the derivatives is greatly improved when we change the proposal from the prior to the uniform distribution over the prevalences.

Appendix C. The mathematical model of LF transmission dynamics

Appendix C.1. TRANSFIL model description

We employed the mathematical model of lymphatic filariasis (LF) transmission TRANSFIL (Irvine et al., 2015) to carry out the analysis in this paper. The model is a stochastic microsimulation of individuals with worm burden, microfilariae (mf) and other demographic parameters in relation to age and exposure to risk. Humans are modelled individually with their own burden of male and female worms denoted by W_i^m and W_i^f, respectively. Mf concentration in the peripheral blood, denoted by M_i, is also modelled

Proposal distribution	ERND	Integrated squared distance ($\times 1000$)	ESS
Beta(2,1)	Distance	0.23734 (0.01742, 1.03220)	338 (222, 473)
	Histogram	0.46996 (0.02757, 1.56949)	335 (252, 453)
	Discrepancy	0.01647 (0.00335, 0.09844)	164 (96, 255)
$U(0,1)$	Distance	0.00408 (0.00157, 0.01799)	1248 (1161, 1330)
	Histogram	0.00214 (0.00175, 0.00292)	1347 (1253, 1429)
	Discrepancy	0.00025 (0.00021, 0.00029)	748 (713, 805)

Table B.1: Integrated squared distance between the two empirical cdfs (multiplied by 1000) and ESS, averaged over 100 replicates under three different empirical Radon-Nikodym derivatives (ERND) and different proposal distributions. The 95% credible intervals are shown in parentheses.

for each individual and according to the number of fertile female worms is increasing as well as decreasing at constant rate. The total mf density in the population contributes towards the current density of L3 larvae in the human-biting mosquito population. Therefore, the model describes the dynamics of individual human, worm inside the host, mf inside the host and larvae inside the mosquito. A detailed description and mathematical formulation of the model are given in Irvine et al. (2015) and more recently in Smith et al. (2017), so here we provide a summary.

Worm dynamics

For each individual i, both male and female worms are added according

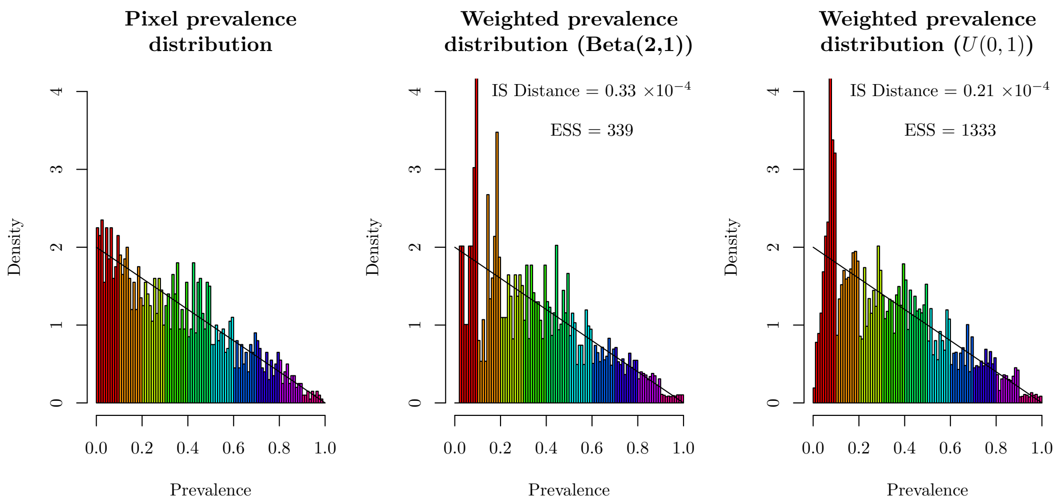

Figure B.6: The estimated weighted prevalence distributions obtained using the histogram-based empirical Radon-Nikodym derivative for 10 bins using a Beta(2,1) (middle panel) and $U(0,1)$ proposal (right panel) is compared to the true pixel prevalence distribution (left panel). Different colours correspond to prevalences in different bins. For reference we also provide the integrated squared (IS) distance and the ESS.

to their bite risk b_i which is individually sampled from a gamma distribution with mean = 1 and shape parameter k. The rate at which an individual i acquires an adult worm, either female or male, is described by the following expression:

$$0.5\lambda b_i(V/H)\psi_1\psi_2 s_2 h(a),$$

where λ is the number of bites per mosquito, V/H is the ratio of vectors to hosts, ψ_1 is the probability that an L3 larvae leaves the host during a biting event, ψ_2 is the probability that the L3 enters the host, s_2 is the proportion of L3 within the host that develop into adult worms and $h(a)$ is the age-dependent biting rate which increases with body

size to saturate at age nine. Finally, we assume that each worm has a constant death rate μ.

Mf dynamics

For an individual i, the dynamics of mf are given by the following expression:

$$\frac{\mathrm{d}M_i}{\mathrm{d}t} = \alpha W_i^f \mathbb{I}(W_i^m > 0) - \gamma M_i,$$

with α being the production rate of mf per worm, γ the constant death rate of mf and function $\mathbb{I}$ is one if there are male worms and zero if not. This expression follows from the fact that *W. bancrofti* is completely polygamous and therefore the mf production rate depends upon the number of female worms combined with the presence of at least one male in the human host.

Larvae dynamics

Larvae development occurs when mf entered the mosquito during a blood meal form an infected host. There are two forms of this relationship depending on the genus of mosquito vectors as expressed below:

$$L(m) = \kappa_{s1}\left(1 - e^{-r_1 m/\kappa_{s1}}\right) \quad \text{for } \textit{Culex},$$
$$L(m) = \kappa_{s2}\left(1 - e^{-r_2 m/\kappa_{s2}}\right)^2 \quad \text{for } \textit{Anopheles},$$

where m is the concentration of mf per 20μL taken during a blood meal and r, κ_s are the parameters which are related to the functional form of the uptake curve (Gambhir and Michael, 2008). The uptake of larvae is an average of mf concentration in the peripheral blood over

all individuals weighted by their bite-risk b_i and is described by the following function:

$$\tilde{L} = \frac{\sum_i L(m_i) b_i}{\sum_i b_i},$$

giving the average number of larvae per mosquito. Using this expression we can calculate the averaged number of larvae taken up in the population $\tilde{L}$ as follows:

$$\frac{\mathrm{d}L}{\mathrm{d}t} = \lambda g \tilde{L} - (\sigma + \lambda \psi_1) L,$$

where λ is the number of bites per mosquito, g is the proportion of mosquitoes which pick up infection when biting an infected host, σ is the death rate of mosquitoes and ψ_1 is the proportion of L3 leaving the mosquito per bite.

Finally the equilibrium value for L3 in a mosquito is given by:

$$L^* = \frac{\lambda g \tilde{L}}{\sigma + \lambda \psi_1}.$$

Host dynamics

Each human begins with zero infection and as mentioned before, has a bite-rate of exposure drawn from a gamma distribution with mean = 1 and shape parameter k. The shape parameter defines how aggregated bites are amongst individuals and consequently defines the aggregation of infection amongst individuals (Irvine et al., 2015). Each individual has a rate of death τ that is assumed to be constant throughout an individuals lifetime with a cut-off at age 100.

A list of the basic model parameter values is provided in Table C.3, with specification of their source. In Table C.4 we provide the parameters that vary spatially across the study area, along with the distribution that are generated from.

Symbol	Definition	Value	Source
λ	Number of bites per mosquito	10 per month	Rajagopalan (1980); Subramanian et al. (1994)
$a_{\max}$	Age at which exposure to mosquitoes reaches its max level	20	Subramanian et al. (2004)
ψ_1	Proportion of L3 leaving mosquito per bite	0.414	Hairston and de Meillon (1968)
ψ_2	Proportion of L3 leaving mosquito that enter host	0.32	Ho and Ewert (1967)
s_2	Proportion of L3 entering host that develop into adult worms	0.00275	Norman et al. (2000); Stolk et al. (2008)
μ	Death rate of adult worms	0.0104 per month	Evans et al. (1993)
α	Production rate of Mf per worm	0.2 per month	Hairston and de Meillon (1968)

γ	Death rate of Mf	0.1 per month	Hairston and de Meillon (1968); Ottesen and Ramachandran (1995)
g	Proportion of mosquitoes which pick up infection when biting an infected host	0.37	Subramanian et al. (1998)
σ	Death rate of mosquitoes	5 per month	Ho and Ewert (1967)
$h(a)$	Parameter to adjust rate at which individuals of age a are bitten	Linear from 0 to 10, with max 1	Norman et al. (2000)
χ_1	Proportion of Mf killed for an individual MDA round using ALB and DEC	0.95	Ismail et al. (1998); Michael et al. (2004)
κ_1	Proportion of adult worm permanently sterilised during MDA round using ALB and DEC	0.55	Ismail et al. (1998); Michael et al. (2004)
ρ	Systematic adherence of MDA	0.35	Stolk et al. (2018)

Table C.3: Description of the basic model parameters that are assumed to be fixed across the study area.

Symbol	Definition	Value
η	Size of population in simulation	Values are draw from the proposal distribution given in Figure C.7(a)
V/H	Ratio of number of vectors to hosts	Values are draw from the joint prior distribution given in Figure C.7(b)
k	Aggregation parameter of individual exposure to mosquitoes	Values are draw from the joint prior distribution given in Figure C.7(b)
α_{Imp}	Importation rate	Values are draw from a uniform prior distribution $\mathcal{U}(0, 0.0005)$
p_C	Coverage of MDA	65% or 80% depending on the future intervention assumptions

Table C.4: Description of the basic model parameters that are assumed to be varied across the study area.

Appendix C.2. Implementation details

To generate the require range of mf prevalences for the seven African countries that we considered, i.e Ethiopia, Sudan, South Sudan, Eritrea, Kenya, Tanzania and Uganda, we varied four parameters of the model; the population size, the vector to host ratio (V/H), the aggregation parameter of individual exposure to mosquitoes (k) and the importation rate (α_{Imp}). More specifically, for the importation rate we used a uniform prior distribution with minimum 0 and maximum 0.0005 (max $\frac{5}{10\,000}$ infections per month). We investigate different maximum values for the importation rate, and we chose the one which give us the desired results without driving the dynamics of the disease. In addition, the interventions reduce the prevalence over time, and therefore as years pass, we decrease the importation rate after intervention in proportion to the reduction in prevalence seen in pilot simulations. More specifically, we produce 2 000 simulations with constant importation rate over five years of MDA. Then, for our main set of simulations, we adjust the importation rate according to how the prevalence changed in our pilot runs after the intervention was applied.

Parameters V/H and k were drawn from a range of plausible values based on previously analysed data (Irvine et al., 2015, 2017; Smith et al., 2017). The graphical representation of their prior distribution is shown in the left panel of Figure C.7. We then generate 100 000 parameter vectors by randomly sampled from these prior distributions of parameters V/H, k and α_{Imp} and from the proposal distribution of the population size as described in Section 4.3 and showed in the right panel of Figure C.7. These samples were then used to generate 100 000 simulations from the model for each scenario specified in

the main text, using the exact years and coverage of the MDA treatments. Our simulations here are focused on areas with anopheles as the dominant vector species. Finally, we set $\delta = 0.01$.

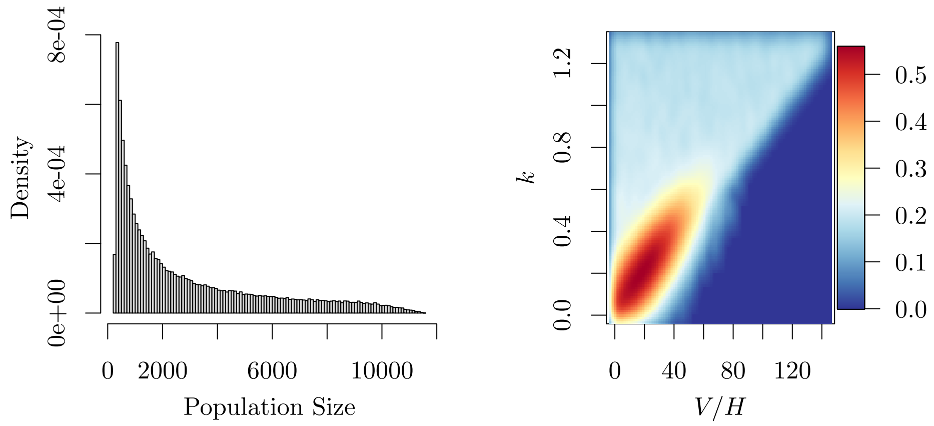

(a) Proposal density of the population sizes.

(b) Joint prior distribution on the vector to host ratio (V/H) and aggregation parameter of individual exposure to mosquitoes (k).

Figure C.7: Distributions assigned to parameters that vary spatially across the study area.

Appendix D. Additional results

In this section we provide additional results for the analysis of the LF data in East Africa of Section 4.3 of the main manuscript. The performance of the method described in Section 2 was assessed by comparing the observed and the estimated number of people at each pixel in the left panel of Figure D.8. In the right panel of Figure D.8, we further evaluated the performance of our method by plotting effective sample size per pixel. Figures D.9 and

D.10 illustrate future predictions of the prevalence under different control scenarios for the first and fifth year.

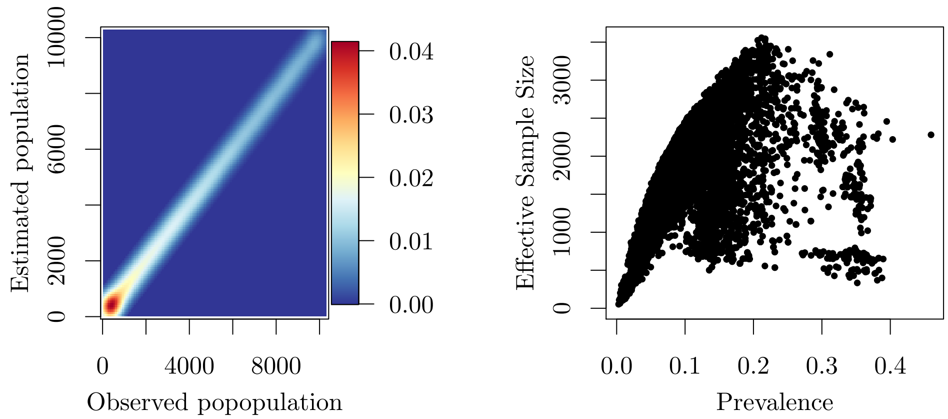

(a) Comparison of the estimated number of people per pixel with the observed value.

(b) Effective sample size against the estimated median prevalence for each pixel.

Figure D.8: Performance assessment of our method.

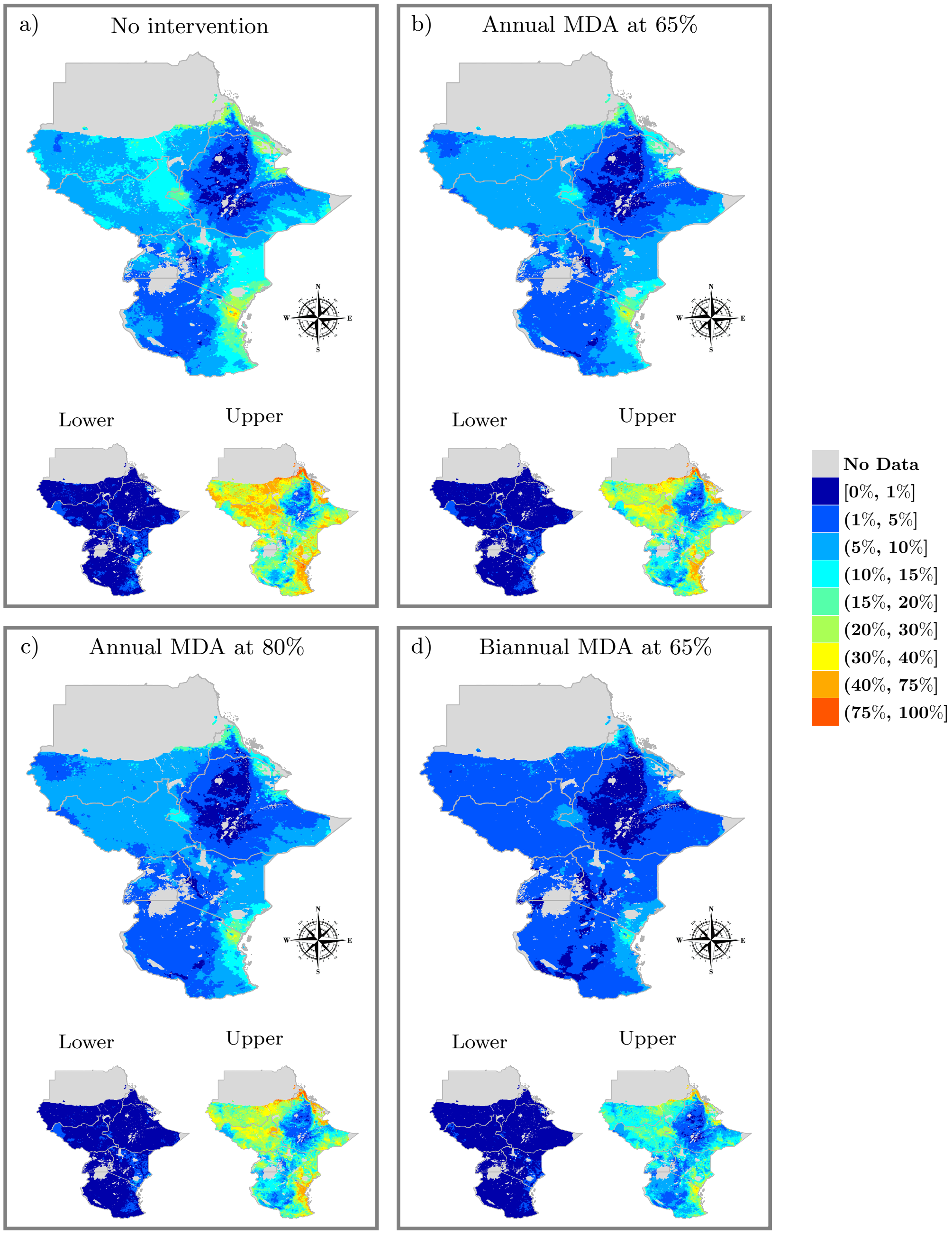

Figure D.9: Prevalence after 1 year under: a) no intervention; annual MDA with coverage of b) 65%; c) 80% and d) biannual MDA at 65% coverage predicted at 5×5 km resolution. Point estimates along with lower (2.5%) and upper (97.5%) percentiles are presented.

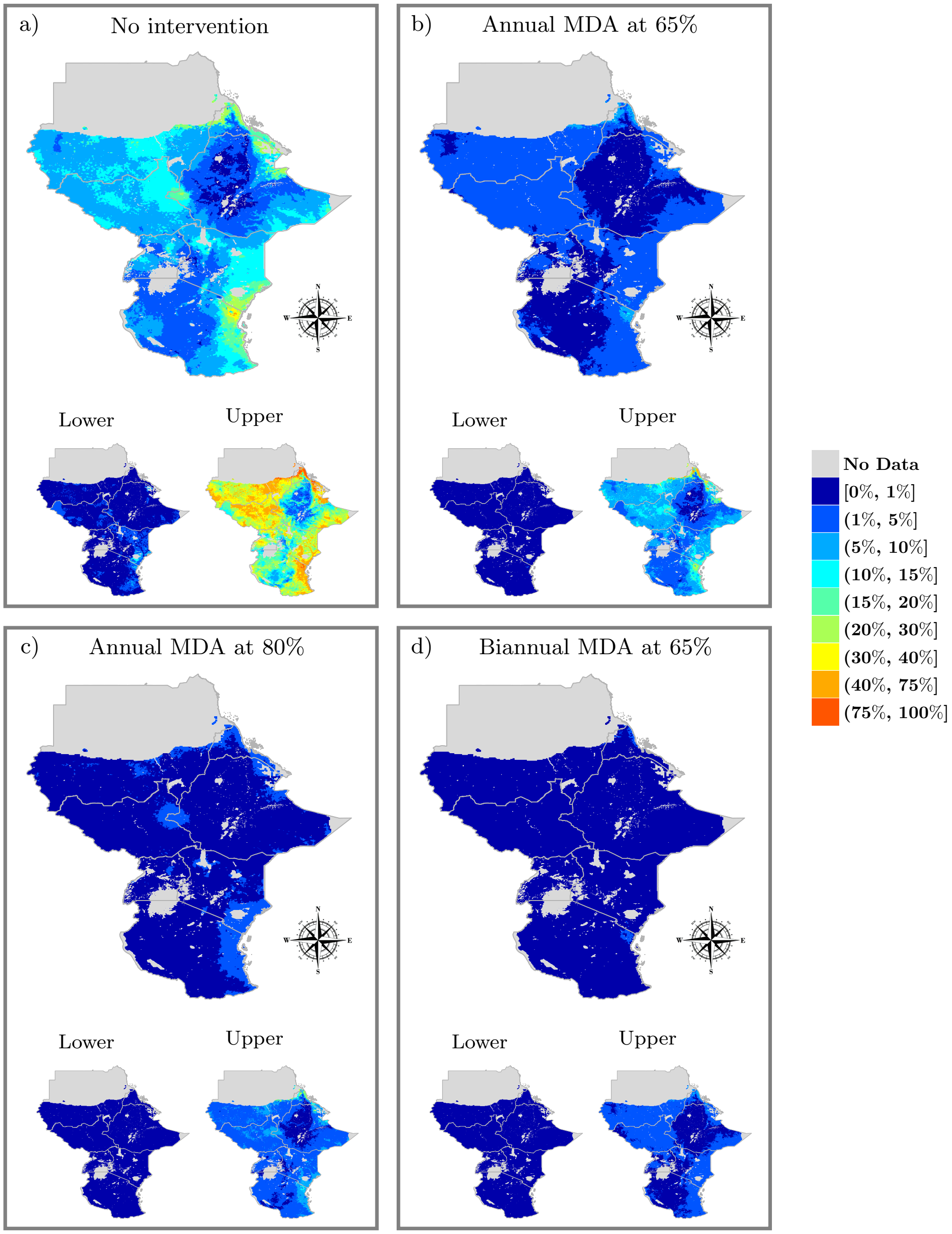

Figure D.10: Prevalence after 5 years under: a) no intervention; annual MDA with coverage of b) 65%; c) 80% and d) biannual MDA at 65% coverage predicted at 5 $\times$ 5 km resolution. Point estimates along with lower (2.5%) and upper (97.5%) percentiles are presented.

References

Evans, D. B., Gelband, H., Vlassoff, C., 1993. Social and economic factors and the control of lymphatic filariasis: a review. Acta Tropica 53 (1), 1–26.

Gambhir, M., Michael, E., 2008. Complex ecological dynamics and eradicability of the vector borne macroparasitic disease, lymphatic filariasis. PLoS One 3 (8), e2874.

Hairston, N. G., de Meillon, B., 1968. On the inefficiency of transmission of Wuchereria bancrofti from mosquito to human host. Bulletin of the World Health Organization 38 (6), 935.

Ho, B. C., Ewert, A., 1967. Experimental transmission of filarial larvae in relation to feeding behaviour of the mosquito vectors. Transactions of the Royal Society of Tropical Medicine and Hygiene 61 (5), 663–666.

Irvine, M. A., Reimer, L. J., Njenga, S. M., Gunawardena, S., Kelly-Hope, L., Bockarie, M., Hollingsworth, T. D., 2015. Modelling strategies to break transmission of lymphatic filariasis - aggregation, adherence and vector competence greatly alter elimination. Parasites and Vectors 8 (1), 547.

Irvine, M. A., Stolk, W. A., Smith, M. E., Subramanian, S., Singh, B. K., Weil, G. J., Michael, E., Hollingsworth, T. D., 2017. Effectiveness of a triple-drug regimen for global elimination of lymphatic filariasis: a modelling study. The Lancet Infectious Diseases 17 (4), 451–458.

Ismail, M. M., Jayakody, R. L., Weil, G. J., Nirmalan, N., Jayasinghe, K. S. A., Abeyewickrema, W., Sheriff, M. H. R., Rajaratnam, H. N., Amarasekera, N. D. D. M., De Silva, D. C. L., et al., 1998. Efficacy of single

dose combinations of albendazole, ivermectin and diethylcarbamazine for the treatment of bancroftian filariasis. Transactions of the Royal Society of Tropical Medicine and Hygiene 92 (1), 94–97.

Michael, E., Malecela-Lazaro, M. N., Simonsen, P. E., Pedersen, E. M., Barker, G., Kumar, A., Kazura, J. W., 2004. Mathematical modelling and the control of lymphatic filariasis. The Lancet Infectious Diseases 4 (4), 223–234.

Norman, R. A., Chan, M.-S., Srividya, A., Pani, S. P., Ramaiah, K. D., Vanamail, P., Michael, E., Das, P. K., Bundy, D. A. P., 2000. EPIFIL: the development of an age-structured model for describing the transmission dynamics and control of lymphatic filariasis. Epidemiology and Infection 124 (3), 529–541.

Ottesen, E. A., Ramachandran, C. P., 1995. Lymphatic filariasis infection and disease: Control strategies. Parasitology Today 11 (4), 129–131.

Rajagopalan, P. K., 1980. Population dynamics of Culex pipiens fatigans, the filariasis vector, in Pondicherry: Influence of climate and environment. Proceedings of the Indian National Science Academy 6, 745–752.

Smith, M. E., Singh, B. K., Irvine, M. A., Stolk, W. A., Subramanian, S., Hollingsworth, T. D., Michael, E., 2017. Predicting lymphatic filariasis transmission and elimination dynamics using a multi-model ensemble framework. Epidemics 18, 16–28.

Stolk, W. A., De Vlas, S. J., Borsboom, G. J. J. M., Habbema, J. D. F., 2008. LYMFASIM, a simulation model for predicting the impact of lymphatic

filariasis control: quantification for African villages. Parasitology 135 (13), 1583–1598.

Stolk, W. A., Prada, J. M., Smith, M. E., Kontoroupis, P., De Vos, A. S., Touloupou, P., Irvine, M. A., Brown, P., Subramanian, S., Kloek, M., et al., 2018. Are alternative strategies required to accelerate the global elimination of lymphatic filariasis? Insights from mathematical models. Clinical Infectious Diseases 66 (Supplement_4), S260–S266.

Subramanian, S., Krishnamoorthy, K., Ramaiah, K. D., Habbema, J. D. F., Das, P. K., Plaisier, A. P., 1998. The relationship between microfilarial load in the human host and uptake and development of Wuchereria bancrofti microfilariae by Culex quinquefasciatus: a study under natural conditions. Parasitology 116 (3), 243–255.

Subramanian, S., Manoharan, A., Ramaiah, K. D., Das, P. K., 1994. Rates of acquisition and loss of Wuchereria bancrofti infection in Culex quinquefasciatus. The American Journal of Tropical Medicine and Hygiene 51 (2), 244–249.

Subramanian, S., Stolk, W. A., Ramaiah, K. D., Plaisier, A. P., Krishnamoorthy, K., Van Oortmarssen, G. J., Amalraj, D. D., Habbema, J. D. F., Das, P. K., 2004. The dynamics of Wuchereria bancrofti infection: a model-based analysis of longitudinal data from Pondicherry, India. Parasitology 128 (5), 467–482.
